# Pentagalloyl Glucose and Cisplatin Combination Treatment Exhibits a Synergistic Anticancer Effect in 2D and 3D Models of Head and Neck Carcinoma

**DOI:** 10.3390/ph15070830

**Published:** 2022-07-04

**Authors:** Jiraporn Kantapan, Nuttawadee Intachai, Nopawit Khamto, Puttinan Meepowpan, Padchanee Sangthong, Kittichai Wantanajittikul, Nathupakorn Dechsupa, Imjai Chitapanarux

**Affiliations:** 1 Molecular Imaging and Therapy Research Unit, Department Radiologic Technology, Faculty of Associated Medical Sciences, Chiang Mai University, Chiang Mai 50200, Thailand; jiraporn.kan@cmu.ac.th; 2 Department Radiologic Technology, Faculty of Associated Medical Sciences, Chiang Mai University, Chiang Mai 50200, Thailand; nuttawadee.i@cmu.ac.th (N.I.); kittichai.wan@cmu.ac.th (K.W.); 3 Department of Chemistry, Faculty of Science, Chiang Mai University, Chiang Mai 50200, Thailand; nopawit_k@cmu.ac.th (N.K.); pmeepowpan@gmail.com (P.M.); padchanee.sangthong@cmu.ac.th (P.S.); 4 Graduate School, Chiang Mai University, Chiang Mai 50200, Thailand; 5 Center of Excellence in Material Science and Technology, Chiang Mai University, Chiang Mai 50200, Thailand; 6 Department of Radiology, Division of Radiation Oncology, Faculty of Medicine, Chiang Mai University, Chiang Mai 50200, Thailand

**Keywords:** pentagalloyl glucose, combination therapy, cisplatin, head and neck cancer, signal transducer and activator of transcription 3, synergic effects, apoptosis, anticancer, chemosensitizer, tumor spheroids

## Abstract

Although cisplatin is a first-line chemotherapy drug for head and neck squamous cell carcinoma (HNSCC), its therapeutic efficacy is limited owing to serious side effects and acquired drug resistance. This study determined whether combining pentagalloyl glucose (PGG) and cisplatin enhanced their anti-tumor activities on HNSCC cell lines. We investigated the anticancer effect of PGG combined with cisplatin in 2D and 3D multicellular spheroid cell culture. The results revealed that PGG combined with cisplatin inhibited cell viability and produced synergistic effects. PGG potentiates the anticancer effect of cisplatin by promoting apoptosis and inhibiting cell migration. The western blot and molecular docking analysis revealed that the synergistic effect of the combination treatment may be related to the PGG-mediated reduced expression of phosphorylated STAT3 and phosphorylated Akt. Furthermore, we found that the combined treatment of PGG and cisplatin’s effect on 3D multicellular spheroid size was more potent than the monotherapies. Our findings indicated that the combination therapy of PGG and cisplatin synergistically inhibited HNSCC cancer cell viability and induced apoptosis in 2D and 3D models. The present results suggested that PGG may be a promising adjunct drug used with cisplatin for a practical therapeutic approach to head and neck cancer.

## 1. Introduction

Head and neck squamous cell carcinoma (HNSCC) is the sixth most common cancer worldwide and has a poor prognosis with a high mortality rate [1]. Locoregional recurrence at primary and regional lymph nodes due to treatment resistance is the main cause of morbidity and the contributing factor to poor survival outcome of HNSCC. Despite advances in therapeutic approaches, including minimally invasive surgery, precisely targeted radiotherapy, chemotherapy, and monoclonal antibody therapy, only a slight improvement has been achieved in the overall survival rates for HNSCC patients over the last two decades [2]. Cisplatin is a well-known chemotherapeutic drug used to treat various solid tumors, including head and neck squamous cell carcinoma. Its mode of action has been linked to its ability to crosslink with the purine bases on the DNA, leading to DNA damage and impairing DNA repair mechanisms, subsequently inducing apoptosis in cancer cells [3]. Combining radiotherapy with cisplatin is the standard treatment for HNSCC. Because of the radio-sensitizing role of cisplatin, concurrent radiotherapy and cisplatin significantly improve local and regional control rates [4]. However, this regimen is associated with developing chemotherapeutic drug resistance and severe toxicities with devastating effects, including kidney toxicity, oral mucositis, decreased immunity to infections, and gastrointestinal disorders, which significantly narrow the therapeutic ratio and limit the clinical usage [5,6]. Therefore, there is an urgent need to develop new treatments that include more effective and less toxic novel targets for improving the clinical outcome of this fatal disease.

**Figure 1:**
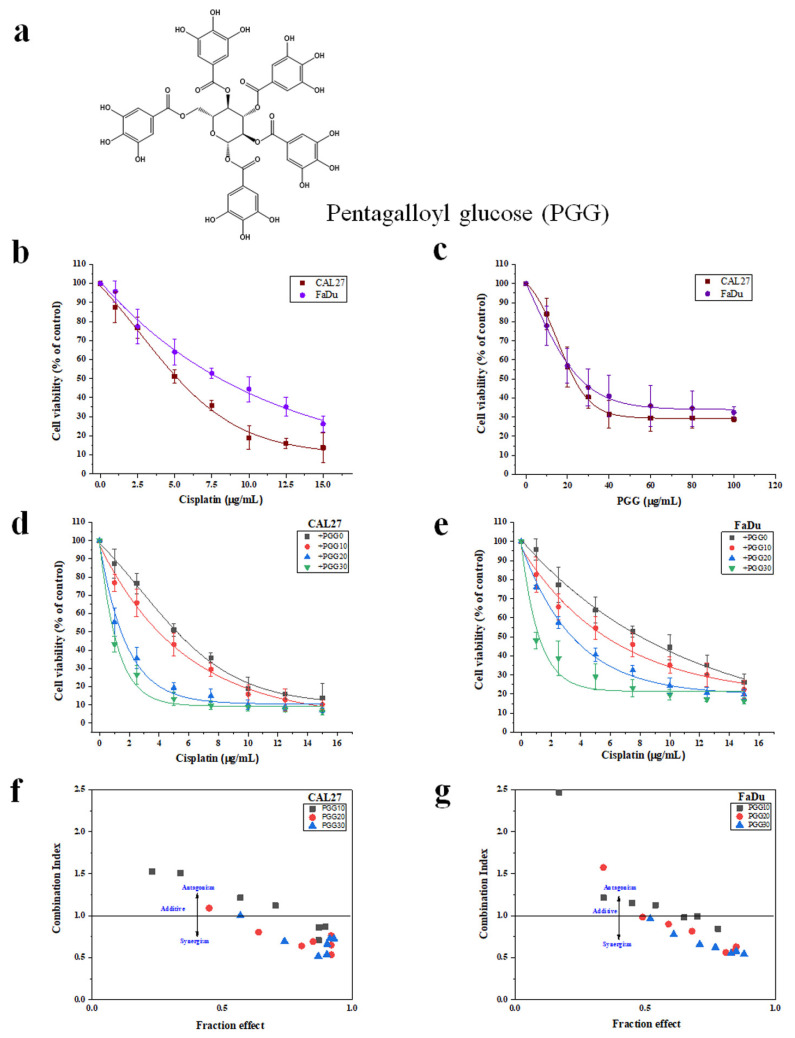
The cell proliferation inhibitory effect of PGG and cisplatin in monotherapy and in combination on HNSCC cancer cells in the 2D culture determined by the MTT assay. (**a**) Chemical structure of pentagalloyl glucose (PGG). (**b**,**c**) A dose–response curve of the cytotoxic effect of cisplatin (**b**) and PGG (**c**) alone on CAL27 and FaDu cell viability after 48 h treatment. (**d**,**e**) Effect of the combination therapy of 10, 20, and 30 µg/mL PGG and serial concentrations of cisplatin on CAL27 (**d**) and FaDu (**e**) cell viability after 48 h treatment, determined by MTT assay. (**f**,**g**) Fa-CI plot analysis of the combination treatment of 10, 20, and 30 µg/mL PGG and cisplatin on CAL27 and FaDu cell viability. PGG, pentagalloyl glucose; Fa, fraction effect; CI, combination index.

Because of the molecular complexity of cancer, a treatment modality that combines two or more therapeutic agents with different mechanisms of action, so-called combination therapy, has proven more effective in successful treatment [7]. It can target critical pathways in a synergistic or additive manner. This approach potentially reduces drug resistance and the chemotherapeutic drug doses in the regimen, helping to circumvent the severe side effects while providing anti-cancer benefits [7,8]. Increasing evidence has suggested that plant polyphenols might be combined with conventional therapy (chemo/radiotherapy) to sensitize tumor cells to the treatment by inhibiting the pathway that leads to treatment resistance [9,10,11]. Pentagalloyl glucose (1,2,3,4,6-Penta-*O*-galloyl-β-D-glucose, PGG; molecular weight = 940.67 g/mol) (illustrated in Figure 1a), a naturally occurring polyphenolic water-soluble gallotannin, is the main biologically active metabolite found, isolated, and purified in *Bouea macrophylla* [12,13]. PGG exhibits anti-cancer and anti-metastasis activity toward several types of cancer cells, including breast [14,15], leukemia [16], HNSCC [13], and prostate [17,18]. The anti-cancer mechanisms of PGG are different from those of chemotherapeutic drugs. Its anti-cancer effect is strongly associated with the inactivation of the signal transducer and activator of transcription 3 (STAT3). Lee et al. reported that PGG inhibited triple-negative breast cancer (TNBC) growth and metastasis. In this study, PGG exerted anti-tumorigenic effects by inhibiting STAT3 activation, which further inhibited angiogenesis and proliferation, and induced apoptosis in TNBC [15]. Additionally, PGG inhibits STAT3 phosphorylation, resulting in the downregulation of downstream target Bcl-XL and Mcl-1, induces caspase-mediated apoptosis in prostate cancer cells in vitro, and decreases in vivo tumor xenograft growth [19]. Our previous study showed that pretreatment with Maprang (*Bouea macrophylla*) seed extract (MPSE), which contains PGG as an active compound, before irradiation of breast cancer cells inhibits the radiation-induced epithelial-to-mesenchymal transition (EMT) process and confers radiosensitivity [20]. In addition, Kantapan et al. recently reported that PGG isolates from the extraction of *Bouea macrophylla* seeds suppress tumor-sphere formation and decrease the protein expression of cancer stem cell markers in the HNSCC cell line [21]. Interestingly, PGG has been shown to enhance the sensitization to radiotherapy and chemotherapy and enhance the anti-cancer effect in renal and hepatocellular cancer cells [21,22]. Considering its sensitizing potential and relatively low toxicity profile, we wished to test PGG as a chemosensitizing drug for cisplatin chemotherapy in HNSCC cells. Using the 2D and 3D tumor models with HNSCC cell lines, we aimed to determine whether the combination of cisplatin and PGG isolated from *Bouea macrophylla* seeds resulted in an enhancement of their in vitro (2D and 3D cultured) anti-tumor activities on HNSCC cell lines compared to the single compounds. Moreover, we also evaluated the molecular mechanisms of PGG-induced STAT3 pathway inactivation by protein expression assay. The molecular interactions between PGG and STAT3 were further investigated in silico using molecular docking, molecular dynamic simulation, and Molecular Mechanics Poisson–Boltzmann Surface Area (MM-PBSA) approaches. In the present study, we found that in the 2D and 3D cultures of HNSCC cells, the combination of cisplatin and PGG exerted a synergistic cytotoxic effect and provided experimental evidence that the mechanism of their synergism was related to PGG-induced inactivation of STAT3.

## 2. Results

### 2.1. The Combination of PGG with Cisplatin Synergistically Inhibits Cell Proliferation and Clonogenic Survival of CAL27 and FaDu Cells in 2D Culture

**Figure 2:**
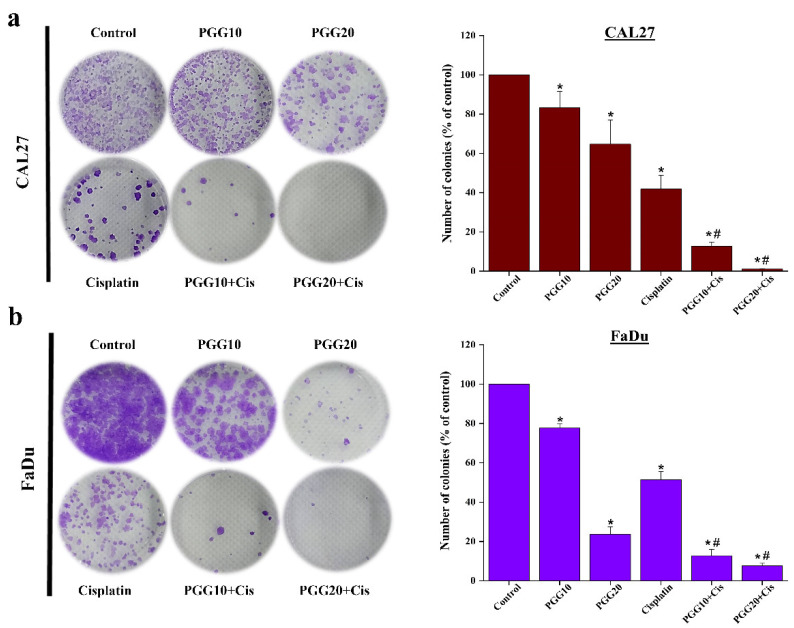
PGG enhances the role of cisplatin in survival of HNSCC cancer cells. Clonal survival analysis showed the number of colonies of CAL27 (**a**) and FaDu (**b**) cells after treatment with PGG (10, 20 µg/mL), cisplatin (2 µg/mL), or the combination of two drugs. Representative images are shown in the left panel, and quantification graphs are shown in the right panel. Data are expressed as (mean ± SD) of three independent experiments. * *p* < 0.05: significantly different from control, and # *p* < 0.05: significantly different from PGG and cisplatin treatment alone. PGG, pentagalloyl glucose; Cis, cisplatin.

To determine whether PGG enhanced the efficacy of cisplatin, the cytotoxicity of PGG and cisplatin to CAL27 and FaDu HNSCC cells in 2D cultures was investigated by applying both compounds individually and in combination. We first determined the appropriate experimental doses for PGG and cisplatin in HNSCC cell lines. PGG inhibited the proliferation of CAL27 and FaDu cells in a concentration-dependent manner with IC_50_ values of 23.7 ± 2.3 and 29 ± 5.3 µg/mL, respectively. Moreover, PGG has less cytotoxicity toward normal fibroblast L929 cells, as evidenced by the IC_50_ of more than 100 µg/mL at 48 h of incubation time (Supplementary Figure S1). Cisplatin showed IC_50_ values of 5.4 ± 0.4 and 7.6 ± 1.4 µg/mL against CAL27 and FaDu cells, respectively (Figure 1b,c). Doses of the combination treatment were selected based on their cytotoxic effects in monotherapy, and only concentrations above IC_50_ were included in the study. Next, we tested the selected doses in the non-constant ratio drug model study on both cell lines. The combination treatment significantly inhibited the proliferation of both cell lines in a dose-dependent manner (Figure 1d,e). The growth inhibitory rates of cisplatin were significantly increased in the presence of 10, 20, and 30 µg/mL PGG, with the IC_50_ of cisplatin reduced from 5.4 ± 0.4 to 4.0 ± 0.1, 1.4 ± 0.2, and 0.8 ± 0.6 µg/mL, respectively, for CAL27 cells and reduced from 7.6 ± 1.4 to 5.4 ± 0.9, 3.6 ± 1.7, and 1.2 ± 0.6 µg/mL, respectively, for FaDu cells (Table 1). The Chou–Talalay combination index (CI) revealed that the combined PGG and cisplatin treatment resulted in even antagonist, additive, and synergistic effects, depending on the concentration of PGG. When PGG concentrations at 10 µg/mL were combined with any tested cisplatin doses (2.5–15 µg/mL), only additive or antagonist effects were observed in FaDu cells, while with high concentrations (12.5 and 15 µg/mL) of cisplatin, a synergistic response was achieved in CAL27 cells. In contrast, 20 or 30 µg/mL of PGG combined with any tested doses of cisplatin caused a synergistic cytotoxic effect in both cell lines (Figure 1f,g). The CI values at different concentrations of PGG determined at 50% cell kill exhibited either an antagonist or synergistic effect between PGG and cisplatin against both HNSCC cell lines. The combinations exhibited synergism in both cell lines with PGG at 20 and 30 µg/mL and CI values at 0.64 and 0.51, respectively, in CAL27 cells and CI values of 0.81 and 0.62, respectively, in FaDu cells. Furthermore, the dose reduction index (DRI) exhibited considerable dose reduction for cisplatin owing to synergistic behavior; in CAL27 cells, the IC_50_ of cisplatin was decreased 1.35-, 3.86-, and 6.75-fold for PGG at 10, 20, and 30 µg/mL, respectively. FaDu cells were reduced 1.4-, 2.1-, and 6.33-fold for PGG at 10, 20, and 30 µg/mL, respectively (Table 1). The dose reduction levels were specific to each cell line, considering that the effect of the combination therapy was more potent in the CAL27 than in the FaDu cell line. Next, we evaluated the combined effects of PGG and cisplatin on cell survival using colony-formation assays. In the CAL27 and FaDu cells, both cisplatin and PGG inhibited colony formation, while the combined treatments significantly suppressed colony formation (Figure 2).

### 2.2. PGG Enhanced the Apoptosis Induction Effect of Cisplatin in CAL27 and FaDu Cells

**Figure 3:**
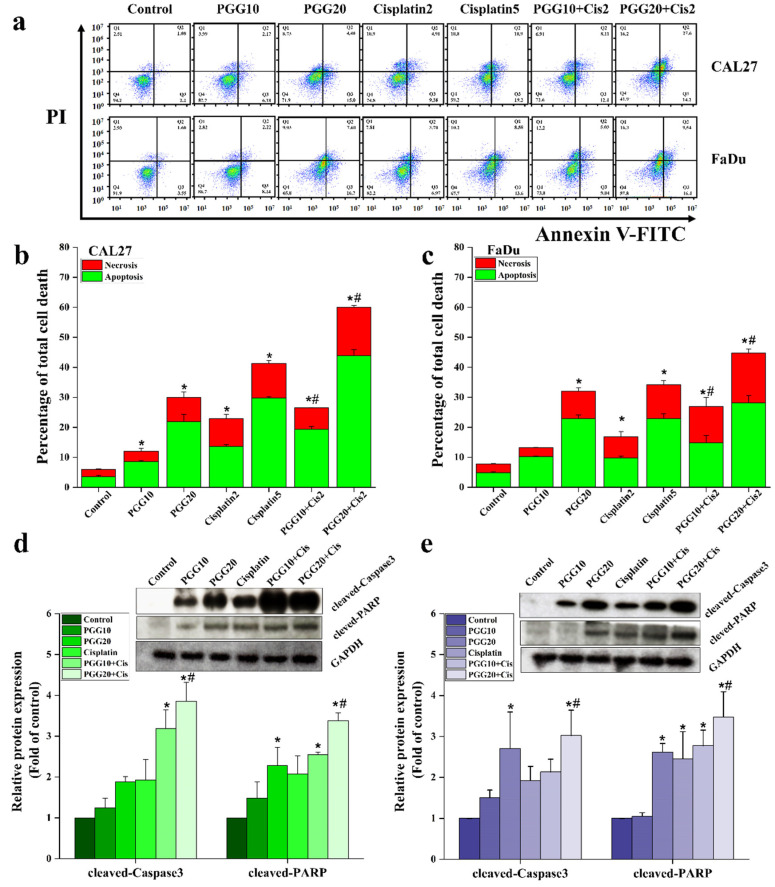
Effect of PGG and cisplatin monotherapy or in combination therapy on apoptosis of HNSCC cancer cells. (**a**) Representative dot plots represent apoptosis responses to therapy with the indicated compound(s) for CAL27 cells and FaDu cells. The quantification data represent the percentage of total cell death in (**b**) CAL27 and (**c**) FaDu cells determined by flow cytometry. Western blot analysis of apoptosis marker, caspase-3, and PARP, the relative density values of related proteins were quantified, and their protein levels were normalized to the loading control GAPDH for (**d**) CAL27 and (**e**) FaDu cells (uncropped images are provided in the Supplementary Figures S4 and S5). All data are expressed as (mean ± SD) of three independent experiments. * *p* < 0.05: significantly different from control, and # *p* < 0.05: significantly different from PGG and cisplatin treatment alone. PGG, pentagalloyl glucose; Cis, cisplatin; PARP, poly ADP ribose polymerase.

Next, we further assessed whether the mechanisms underlying the synergistic interaction of PGG and cisplatin in cellular growth inhibition were related to apoptosis. Double staining annexin V-FITC/PI apoptosis detection was performed to compare control, PGG alone, cisplatin alone, and combined PGG and cisplatin groups by flow cytometry. We chose two doses at approximately IC_20_ and IC_50_ (2 and 5 µg/mL) of cisplatin for apoptosis induction experiments based on the above results. PGG and cisplatin as single drug treatments induced cellular apoptosis in a dose-dependent manner in CAL27 and FaDu cells. Notably, treatment of cancer cells with a sub-lethal dose of cisplatin (2 µg/mL) resulted in only a mild apoptosis rate while, when combined with PGG, a sub-lethal dose of cisplatin significantly increased apoptosis in both cell lines compared to the single treatment (Figure 3a–c). The enhanced apoptosis of the combined treatment was approximately 3-fold higher than 2 µg/mL cisplatin alone and appeared to be higher than treatment with a high concentration of cisplatin (5 µg/mL). Thus, these results demonstrated that cisplatin synergizes with PGG to promote potent apoptosis in HNSCC CAL27 and FaDu cells. Furthermore, we could reduce the dose of the chemotherapeutic drug while preserving the anti-tumor activity by using it in combination with PGG. Then, we explored the mechanism of the combined PGG + cisplatin treatment promoting apoptosis from the molecular level. Specifically, we investigated the expression of apoptotic-related proteins caspase-3 and its downstream factor poly ADP ribose polymerase (PARP), which is the hallmark of apoptosis induction. In this study, we selected only the sub-lethal dose of cisplatin (2 µg/mL) to test its effects in combination with PGG. We selected 10 and 20 µg/mL PGG to sensitize the sub-lethal dosage of anticancer drug-induced apoptosis. We could not collect protein from the high concentration of PGG (30 µg/mL) group owing to the cells’ near-death. The Western blot results showed that the sub-lethal dose of cisplatin treatment partially activated caspase-3 and PARP cleavage expression levels in CAL27 and FaDu cells. On the other hand, when combined with cisplatin, PGG significantly enhanced the apoptotic induction ability of sub-lethal dosage cisplatin (Figure 3d,e), which strongly suggested that PGG can enhance the anti-tumor effect of cisplatin by promoting apoptosis.

### 2.3. PGG Inhibits Migration and Promotes the Anti-Migration Effect of Cisplatin in CAL27 and FaDu Cells

**Figure 4:**
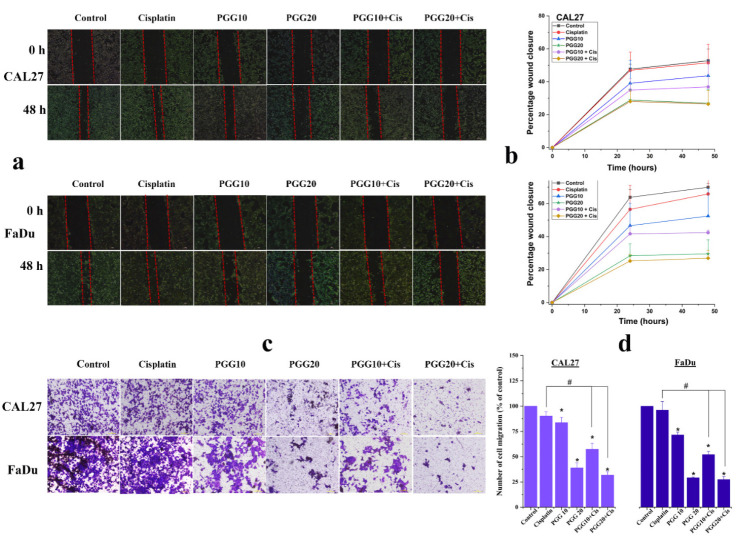
Effects of PGG combined with cisplatin on the migration and invasion of HNSCC cancer cells. Representative microscopic images of wound healing (**a**) and quantification of the percentage of wound closure (**b**) showing the effect of PGG (10, 20 µg/mL) or cisplatin (2 µg/mL) alone, or PGG and cisplatin combined treatment. (**c**) Transwell chamber images of cell migration after treatment with PGG (10, 20 µg/mL) or cisplatin (2 µg/mL) alone or a combination of PGG and cisplatin. Scale bar = 100 µm. (**d**) The quantitative number of migrant cells. All data are expressed as the mean ± SD of three independent experiments. * *p* < 0.05: significantly different from control, and # *p* < 0.05: significantly different from PGG and cisplatin treatment alone. PGG, pentagalloyl glucose; Cis, cisplatin.

The effects of PGG combined with cisplatin on the cancer cell migration of CAL27 and FaDu cells were evaluated by scratch wound healing and Transwell migration assays. As shown in Figure 4a,b, the scratch wound-healing assay showed that cells migrated more quickly to heal the open wound area in the control and sub-lethal dose of cisplatin (2 µg/mL) groups, indicating that a sub-lethal dose of cisplatin did not affect the wound healing ability of CAL27 and FaDu cells. Meanwhile, 20 µg/mL PGG effectively suppressed the migration of cells toward the scratch wound, leaving a large gap area of the open wound. In addition, we found no significant difference after 10 µg/mL PGG treatment alone, but the combination of 10 or 20 µg/mL PGG with 2 µg/mL cisplatin enhanced the inhibitory effects on the migration of CAL27 and FaDu cells compared to the control or cisplatin alone group. This result indicated that combined PGG plus cisplatin treatment enhanced the inhibitory effects on the migration of CAL27 and FaDu cells. Furthermore, the Transwell migration assay results showed a similar trend for 10 and 20 µg/mL PGG combined with 2 µg/mL cisplatin, which significantly inhibited the invasion of CAL27 and FaDu cells compared to the control group or cisplatin-only group (Figure 4c,d). The results further confirmed that PGG combined with cisplatin could inhibit the migration and invasion of CAL27 and FaDu cells. Nevertheless, we found that the combination of 20 µg/mL PGG and 2 µg/mL cisplatin was excellent in delaying wound healing and inhibiting cell migration, but the difference between 20 µg/mL PGG monotherapy was not statistically significant.

### 2.4. Combined Treatments of PGG and Cisplatin Abolished STAT3 and Akt Activation and Enhanced Drug Sensitivity of HNSCC Cells

**Figure 5:**
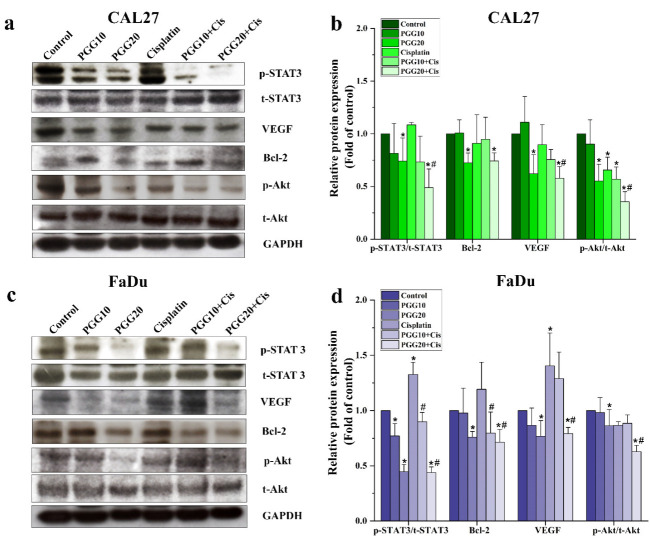
The effect of PGG and cisplatin alone or in combination on STAT3/Akt signaling proteins in HNSCC cancer cells. Representative images from Western blot analysis of the expression of p- STAT3, total STAT3 and p-AKT, total Akt, Bcl-2, VEGF and GAPDH following treatment with either PGG (10, 20 µg/mL) or cisplatin (2 µg/mL) alone, or their combination for 48 h in CAL27 cells (**a**) and FaDu cells (**c**)(uncropped images are provided in the Supplementary Figures S6 and S7). The bar diagram showing the fold change of protein expressions. The relative density values of related proteins were quantified, and their protein levels were normalized to the loading control GAPDH (**b**,**d**). All data are expressed as the mean ± SD of three independent experiments. * *p* < 0.05: significantly different from the control, and # *p* < 0.05: significantly different from cisplatin treatment alone. PGG, pentagalloyl glucose; Cis, cisplatin; Akt, serine/threonine kinase; STAT3, signal transducer and activator of transcription 3; p, phosphorylated; t, total; GAPDH, glyceraldehyde 3-phosphate dehydrogenase; VEGF, vascular endothelial growth factor.

Previous studies have demonstrated that STAT3 plays an important role in tumorigenicity and drug resistance and is aberrantly expressed in HNSCC cancer cells [23,24]. We evaluated whether cisplatin-induced STAT3 activity could be reversed by PGG and could increase the cell sensitivity to cisplatin. We found that both CAL27 and FaDu cells exhibited high levels of phosphorylated STAT3 expression, and treatment with cisplatin for 48 h resulted in a slight increase in phosphorylated STAT3. Moreover, cells treated with PGG alone markedly suppressed the phosphorylation of STAT3 at Tyr705 in a dose-dependent manner, while no apparent changes in total levels of STAT3 were observed in both CAL27 and FaDu cells. The combination of PGG plus cisplatin had an additive effect and reduced STAT3 phosphorylation. Importantly, we found that cisplatin-stimulated active STAT3 was dramatically inhibited by the combination treatment with PGG at 20 µg/mL (Figure 5). Furthermore, the expression of Bcl-2 and VEGF, a critical mediator in cell survival and migration and a downstream target of STAT3, were also decreased in the PGG and the PGG plus cisplatin treatment groups compared to cisplatin alone. Akt is a well-known serine/threonine kinase that plays a crucial role in cell proliferation, survival, and migration [25]. Since PGG and cisplatin inhibited cell proliferation and migration, we wished to determine the Akt phosphorylation status upon these treatments. Phosphorylated Akt was inhibited with the combined treatments of PGG and cisplatin. Total Akt expression levels were not changed (Figure 5). Our data suggested that the PGG and cisplatin combination efficiently inhibited the critical kinase Akt and transcription factor STAT3. These results were consistent with our apoptosis and migration assay, showing reduced cell survival and migration caused by PGG plus cisplatin in both CAL27 and FaDu cells. Since we found that PGG inhibited the phosphorylation of STAT3 in the Western blot analysis, we further elucidated the atomistic binding mechanisms of PGG against the target protein (STAT3) using multiple computational modeling techniques.

### 2.5. Molecular Docking

According to the structure of STAT3, the phosphorylation of STAT3 at the Tyr705 residue is important for dimerization and transactivation. The dimerization of STAT3 occurs through the binding of phosphopeptide-containing pTyr705 from one monomer to the Src-homology 2 (SH2) domain of another monomer [26]. The key amino acid for peptide binding affinity was located at Arg609. This residue contributed to the binding between STAT3 and the phosphorylated peptide [27]. Therefore, compounds that can be bound at the SH2 domain and interact with Arg609 will prevent the dimerization of STAT3, leading to inhibition of the STAT3 signaling pathway. Herein, we applied molecular docking to dock PGG to the SH2 domain of STAT3 to clarify the inhibition of this compound. The validation of the docking protocol was achieved by redocking of a co-crystalized ligand to the active site at the SH2 domain, which showed a binding energy value of –8.6 kcal/mol. The interactions of the docked and co-crystallized ligands were identical. Superimposition of docked and co-crystallized ligands indicated an RMSD value of 0.887 Å (see Supplementary Figure S2). The redocking of the ligand showed the very low RMSD values, indicating good validation of docking protocols. PGG was docked to the SH2 domain of STAT3 with a binding energy value of −6.6 kcal/mol. The interactions generally comprised hydrogen bonding and van der Waals interactions. PGG showed the hydrogen bonding interaction with the key residue Arg609 and other residues in the SH2 domain. For the binding poses and protein-ligand interactions from the molecular docking see Supplementary Figure S3.

### 2.6. Molecular Dynamic Simulation

**Figure 6:**
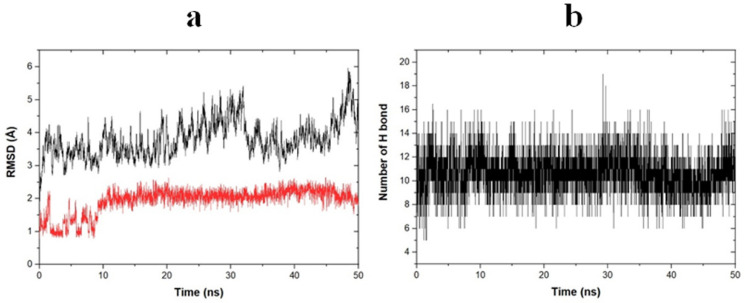
(**a**) Root mean square deviations (RMSDs) of protein backbone in black and ligand in red and (**b**) number of hydrogen bonds between protein and ligand with respect to 50 ns molecular dynamic simulation.

**Figure 7:**
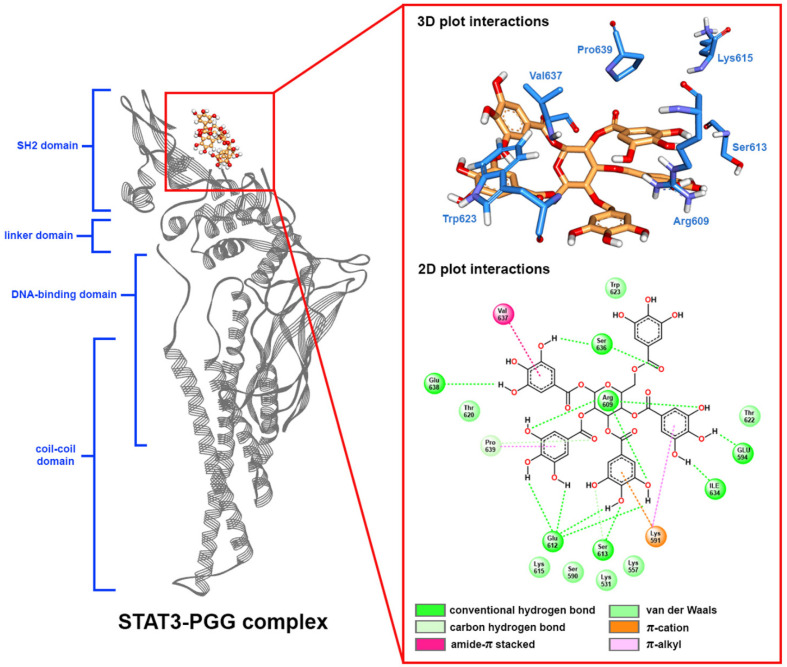
The predicted binding mode in 2-dimension (2D) and 3-dimension (3D) formats of PGG at the binding pocket of STAT3 was obtained from the last frame of the 50 ns molecular dynamic simulation.

The binding pose from molecular docking further underwent molecular dynamic simulation to establish the stability, flexibility, and conformation changing of the protein-ligand complex. In this study, the dynamic of the PGG-STAT3 complex was simulated for 50 ns. The protein-ligand complex stability was evaluated by calculating the root mean square deviation (RMSD) of both protein and ligand, as depicted in Figure 6a. As a result, PGG bound highly stably to the SH2 domain of STAT3. The backbone of STAT3 became stable around 3 ns after simulation and remained stable thereafter with the RMSD values ranging from 3 to 5 Å compared with the initial structure. The PGG became stable around 10 ns and remained stable during the simulation with an RMSD value of around 2 Å. Based on the RMSD values, the fluctuation of both ligand and protein was sightly (RMSD < 2 Å) indicative of the high stability of the protein-ligand complex. The binding pose in the last frame of the molecular dynamics simulation of the STAT3–PGG complex is depicted in Figure 7.

According to the data of the SH2 domain amino acid, most amino acids constructed in the binding site were polar amino acids, including Arg609, Ser61, Glu612, Ser613, Thr620, Ser636, Val637, Glu638, Pro639, Tyr640, Gln644, Tyr657, Lys658, and Ile659. PGG possessed a great number of hydroxy groups that interacted with these polar amino acids through hydrogen bonding. Analysis of hydrogen bonding between receptor and ligand showed that PGG formed stable hydrogen bonding with STAT3 as averaged around 11 H-bonding (Figure 6b). This ability is assumed to be the abundance of hydrogen bonding that encouraged the stability of the protein-ligand complex. We further investigated the stability of the formed hydrogen bonds by measuring the distance and percentage of occupancy as determined with a distance cut-off value of 3.5 Å. The stably formed hydrogen bonds are shown in Table 2. As a result, Glu612 residue played a critical role in hydrogen bonding interaction. This residue contributed four hydrogen bonds, with the percentage of occupancy ranging from 80.14 to 92.91%. The distances between H-bond donor and acceptor atoms were quite short, ranging from 1.6 to 1.7 Å. In addition, the residues Ser636 and Glu638 stably formed hydrogen bonding with PGG by reacting with carbonyl and hydroxy groups. The key amino acid Arg609 formed moderate stable hydrogen bonds with PGG. The interactions occurred when the amino group of guanidine moiety on Arg609 reacted with an oxygen atom of the hydroxy group on PGG at a very short bond distance. However, some hydrogen bonds were unstable, with a percentage of occupancy less than 1%, such as in Glu594 and Ser613. Consequently, a great number of formed hydrogen bonds influenced the strong binding between STAT3 and PGG, and the interaction on the Arg609 residue prevented the binding of phosphorylated protein, leading to inhibition of the STAT3 signaling pathway.

### 2.7. Free Binding Energy Calculation

The free binding energy of STAT3 in complex with PGG was calculated using the MM-PBSA methodology. The last 10 ns of trajectory with snapshots every 10 ps from the molecular dynamic simulation was used for calculation. This method utilized the dynamic of the protein-ligand complex to calculate the binding energy, which was more reliable than the single conformation-based score from the molecular docking technique. The free binding energy with its components, including van der Waals (ΔE_vdw_), electrostatic (ΔE_elect_), polar solvation (ΔE_polar_), and solvent-accessible surface area (SASA) or (ΔE_SASA_), are depicted in Table 3. The binding energy (ΔG_bind_) was −27.938 ± 5.224 kcal/mol and was contributed predominantly by van der Waal and electrostatic interactions with energy values of −46.858 ± 4.360 and −47.356 ± 4.681, respectively.

Analysis of the free energy contribution per residue revealed the amino acids that played a crucial role in the binding of PGG at the SH domain of STAT3. The free binding energy per residue and its 12 major amino acid components are shown in Table 4. As a result, PGG bound tightly with Lys531, Lys557, Arg609, Ser613, Lys615, Thr620, Thr622, Trp623, Ile634, Ser636, Val637, and Pro639 residues. The ΔE_MM_ contributed favorably, resulting in an overall binding of PGG on STAT3, except on Lys531 and Arg609. Polar solvation energies (ΔE_polar_) were mostly unfavorable for binding except on Lys531, which contributed a predominantly negative energy value indicating the easy solvation of PGG. The residues Lys557, Ser636, Val637, and Pro639 mainly contributed potential energy (ΔE_MM_). The residue Lys557 promoted ΔE_MM_ = −1.522 ± 0.036 kcal/mol by a van der Waals interaction between the methylene group (CH_2_) with an aromatic ring of PGG. The primary free binding energy residues contributed were Val637 and Pro639 with the binding energies of −1.402 ± 0.014 and −1.372 ± 0.007 kcal/mol, respectively, through the van der Waals interactions with PGG. Residue Val637 interacted with PGG through π-stacked, π-alkyl interactions of carbonyl and isopropyl moieties, respectively. The pyrrolidine ring of Pro639 interacted with the carbonyl group and aromatic ring of PGG through a carbon-hydrogen bond and π-alkyl interaction. In addition, analysis of the binding energy of key amino acid Arg609 showed that these residues demonstrated the favored and negative binding energy of −0.518 ± 0.043 kcal/mol, contributed predominantly by polar solvation energy. All computational simulations supported the inhibition of STAT3 by PGG.

### 2.8. The Combination of PGG and Cisplatin Exerts a Strong Cytotoxic Effect on HNSCC Cancer Cell Lines in the 3D Cell Culture

**Figure 8:**
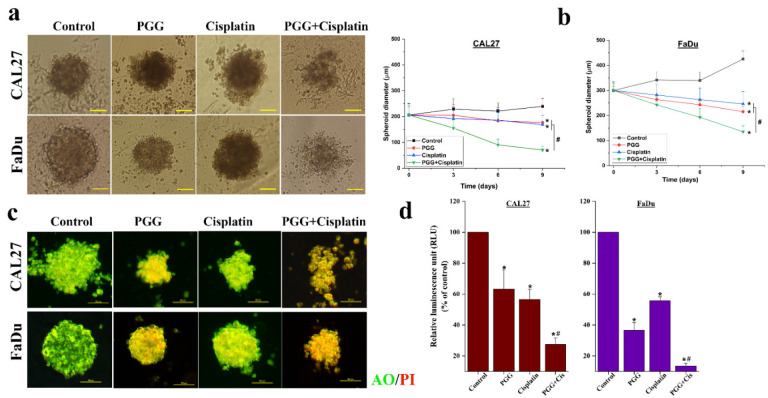
Effects of PGG combined with cisplatin on cell viability in the 3D HNSCC cancer cell culture. The effect of 50 µg/mL PGG, 10 µg/mL cisplatin, and their combination on CAL27 and FaDu multicellular spheroids after 72, 144, and 216 h of treatment. (**a**) Typical images of CAL2 and FaDu multicellular spheroids after 216 h of control or treatment with PGG, cisplatin, and their combination. Scale bar = 100 µm. (**b**) The spheroid size was calculated from three independent experiments (*n* = 3) measuring the size of eight spheroids for each condition. Data are expressed as mean ± SD. (**c**) AO/PI staining revealed the apoptosis induction in 3D spheroids treated with 50 µg/mL PGG, 10 µg/mL cisplatin, and their combination on CAL27 and FaDu multicellular spheroid for 72 h. Scale bar = 100 µm. (**d**) Relative ATP content (% of control) at day 9 of CAL27 and FaDu multicellular spheroid. All data are expressed as the mean ± SD of three independent experiments. * p < 0.05: significantly different from control, and # *p* < 0.05: significantly different from PGG and cisplatin treatment alone. PGG, pentagalloyl glucose; Cis, cisplatin.

We applied the multicellular spheroid technique to determine whether combination therapy exerted its cytotoxic potential in the 3D cell culture; spheroid models were proposed as mimics of the in vivo tumors. Initial spheroid sizes evaluated after 48 h of cell seeding were 200 ± 11 and 300 ± 15 µm (*n* = 100) for CAL27 and FaDu cells, respectively. On the same day, the selected spheroids were treated with (approximately 2IC_50_ of 2D culture system) 50 µg/mL PGG, 10 µg/mL cisplatin, or a combination of PGG and cisplatin. Treatment efficacy was assessed based on spheroid size by measuring at the three different time points (72, 144, and 216 h), and the acridine orange (AO) and propidium iodide (PI) dual-staining assay was used to determine the treatment-induced cell death in the spheroid 72 h after treatment. Moreover, after 9 days of culturing, spheroids were collected and examined for their viability using an ATP determination assay. As expected, the spheroids of both cell lines were less sensitive to drug doses that were effective in the 2D cell culture. Thus, the doses selected for 3D culture were higher than those used in the 2D system. The results revealed that treatment with PGG or cisplatin alone exerts a more negligible effect on tumor spheroids as indicated by the unchanged size in CAL27 cell spheroids. In contrast, in FaDu cell spheroids treatment with PGG or cisplatin alone reduced the growth rate of the spheroids as indicated by the reduced spheroid size, while the combination of PGG with cisplatin resulted in a higher reduction in the spheroid size and cell debris in the surrounding spheroid in both CAL27 and FaDu cell spheroids (Figure 8a,b). Interestingly, FaDu cell spheroids were sensitive to PGG or cisplatin monotherapy, whereas a significant effect was obtained in CAL27 cell spheroids only after combination treatment. The same tendency was observed in the ATP content of the tumor spheroids in the cell viability assay; the combination treatment of PGG with cisplatin led to a significant reduction in the ATP content compared to monotherapy (Figure 8d). In the next step, the effect of treatment-induced cell death in the 3D tumor spheroid was evaluated by AO/PI dual staining. AO is a vital dye that stains both live and dead cells, and PI stains only cells with lost membrane integrity. Therefore, viable cells appeared uniformly green; dead cells incorporated PI and were consequently stained orange. The results are shown in Figure 8c. A total of 50 µg/mL PGG or 10 µg/mL cisplatin treatment slightly induced apoptosis in tumor spheroids as indicated by spheroid nuclei that turned fluorescent orange from the propidium iodide stain, indicating compromised membrane integrity, and these spheroids also exhibited decreased green cytoplasmic fluorescence. The combined PGG and cisplatin treatment resulted in higher cell death induction in tumor spheroids; we observed an increase in isolated single cells and smaller spheroids, indicating the loss of well-circumscribed edges on the otherwise compact spheroids (especially CAL27 cell spheroids). In addition, an increase in the fluorescent intensity of the PI stain resulted in dead cells becoming orange. All indicated apoptosis and necrosis induction in treated tumor spheroids.

## 3. Discussion

This study demonstrated for the first time the ability of PGG to synergize the anti-cancer effects of cisplatin in HNSCC cancer cell lines. Based on the current challenges in HNSCC cancer treatment, cisplatin was combined with radiotherapy in the organ preservation protocol for HNSCC patients. Although the combination of cisplatin and radiotherapy can significantly improve the prognosis of HNSCC patients, the severe side effects of platinum drugs are also a non-negligible fact in clinical applications [6,28]. Therefore, it is essential to find new drugs or novel combination strategies that are more effective against HNSCC and can alleviate the side effects of chemotherapy drugs. An accumulating body of evidence has suggested that therapies having multiple targets result in more dramatic benefits than single-targeted therapies, because each agent has a different target or mechanism of action against cancer cells. Thus, the combination treatment could either enhance the clinical responses while decreasing the drug concentrations required for efficacy, leading to lowering the side effects and the incidence of drug resistance [7].

Combining naturally occurring phytochemicals with chemotherapeutic drugs has received heightened attention. Several compounds from natural products have been reported to improve the therapeutic outcomes of cancer patients when used in combination with chemotherapeutic drugs. Combining these treatments can induce synergetic effects, inhibit side effects, or overcome drug resistance [7,8,11]. Previous studies have suggested the synergistic action of PGG with the traditional chemotherapeutic drug 5-FU in hepatocellular carcinoma. This combination induced apoptosis by increasing the proportion of Bax/Bcl-2 and promoting the activation of caspase-9 and caspase-3. Moreover, the combination also had synergistic effects on aggressive phenotypes of HepG2 cells by downregulating multidrug resistance protein 1 (MDR1) and low-density lipoprotein receptor-related protein 1 (LRP1), suggesting the potential to reverse the resistance to 5-FU of PGG [22]. In this study, we first found that the combination of PGG with cisplatin had a much stronger cell proliferation inhibitory effect on CAL27 and FaDu HNSCC cancer cells in the 2D culture compared with the effect observed in monotherapy experiments. The Chou–Talalay analysis revealed that the drugs acted synergistically. We found that the combination of 20 µg/mL PGG and cisplatin was an effective synergistic dose (CI < 1) (Figure 1f,g and Table 1). This combination reduced the dose of cisplatin required to achieve the same growth inhibitory rate in CAL27 and FaDu cells by 3.9- and 2.1-fold, respectively. Moreover, the dose of 20 µg/mL PGG used in the combination treatment did not induce any cytotoxicity as evaluated on normal mouse fibroblasts L929 cells, indicating that the PGG compound caused cytotoxicity preferentially to cancer cells. Thus, this combination concentration was used in the following experiments. A colony-formation assay was performed to validate the effect over a long-term period. We found that the combination dose of PGG and cisplatin significantly reduced the colony-forming ability of CAL27 and FaDu cells compared to corresponding monotherapy doses and untreated control (Figure 2). Moreover, in this study, we provided evidence that either PGG or cisplatin inhibits the proliferation and induces apoptosis of HNSCC cell lines in a dose-dependent manner. The combination of PGG and cisplatin potentiated the apoptotic effect of cisplatin. A combination sub-lethal dosage of cisplatin (2 µg/mL) and PGG (20 µg/mL) effectively induced apoptosis in HNSCC cell lines. The apoptotic rates obtained with the PGG + cisplatin allowed a reduction in the dose of cisplatin required to achieve the same apoptotic rate in HNSCC cell lines by 2.5 times (Figure 3b,c). The PGG and cisplatin combination compared to cisplatin alone increased PARP proteolytic cleavage and induced the activation of the effector caspase-3, which activated the apoptotic pathways. The model of interaction between PGG and cisplatin, when used in combination in HNSCC cell lines, indicated the onset of the sensitizing effect of the two compounds compared with the associated single treatment after a decrease in their concentrations. Interestingly, the combination treatment with PGG allowed for lowering the dose of cisplatin required to achieve the anti-cancer effect. This will provide an experimental basis for the practical application of PGG in vivo since cisplatin has objectionable side effects such as severe mucositis and nephrotoxicity [28]. Suppressing the growth of HNSCC cells can be achieved using a lower dose of cisplatin when combined with the nontoxic natural product PGG, allowing for reduced toxicity and a vastly improved patient quality of life. In agreement with our result, a previous study revealed the protective effect of PGG against cisplatin-induced cytotoxicity and apoptosis in normal human primary renal epithelial cells. PGG attenuated reactive oxygen species (ROS) production mediated by cisplatin treatment and significantly blocked cisplatin-mediated cytotoxicity [21]. Other studies have shown that PGG inhibits the migration and invasion of several cancer cells [15,18]. Meanwhile, in our study, we found that a combination of PGG and cisplatin can enhance cisplatin’s inhibitory ability in HNSCC cells to migrate and invade. This result was consistent with the study by Ding et al., who reported that a combination of 5-FU and PGG effectively inhibited the migration and invasion of HepG2 cells [22]. However, in this study, we used a sub-lethal concentration of cisplatin (2 µg/mL); at this concentration, we observed little inhibitory effect on the wound healing and migration ability of CAL27 and FaDu cells, as well as this concentration being without any impact on cell killing. Moreover, it has been reported that the cancer cells treated with sub-lethal doses of cisplatin take an adaptive mechanism to escape from the stress condition induced by cisplatin by triggering the epithelial-to-mesenchymal transition (EMT) process and toward increased expression of migratory-related proteins, subsequently facilitating cancer cell migration [29,30]. Interestingly, the combination therapy of sub-lethal dose cisplatin with 10 and 20 µg/mL is more potent for the cell migration inhibition than 2 µg/mL cisplatin monotherapy. An effect likely caused by PGG-mediated suppression of cisplatin-induced EMT process and, consequently, inhibits cell migration, paradoxically in monotherapy with 20 µg/mL PGG seems to have a higher efficacy to impede the migration of both HNSCC cell lines than the combination therapy (Figure 4). This phenomenon may explain that PGG 20 µg/mL indeed affected the inhibition of the rate of cell migration and cell proliferation. In contrast, cisplatin did not, thus indicating a reduction in migration due to an impaired migration and the reduced proliferation effect, which was not the case for combination therapy. The effect of PGG on inhibiting the EMT process has been reported previously. The combination treatment of PGG from Maprang seed extract (MPSE) with radiation can inhibit the radiation-induced EMT process and further confer its radiosensitizing effect [20]. These results encourage the potential of utilizing PGG in combination with conventional therapy to enhance the efficacy of cancer treatment.

HNSCC cells frequently overexpress EGFR and ErbB2 receptors, which activate STAT3 [24,31]. The dysfunctional regulation of the STAT3 oncogene contributes to tumor development and progression in several cancers, including HNSCC [32,33]. The association of STAT3 hyperactivation with poor prognosis, resistance to therapies, and immune escape makes it a tempting target in HNSCC, particularly in HPV-negative HNSCC, where treatment targeting this pathway may be effective [34]. This study chose the HPV-negative HNSCC cell lines, CAL27, and FaDu cells, as a model to test the ability of PGG to synergize the anti-cancer effects of cisplatin. Several studies have demonstrated that PGG can inhibit STAT3 phosphorylation and suppress tumor growth and metastasis in breast and prostate cancer cells [15,18]. Our previous study showed that PGG inhibited the activation of STAT3 in HPV-negative HNSCC cell lines, resulting in the suppressed self-renewal activity of cancer stem cells and inhibiting the tumor spheroid formation. Moreover, the combination treatment with PGG sensitized HPV-negative HNSCC cell lines to the effect of radiation-induced cell death, which may be at least partially mediated by the inhibition of the STAT3 pathway [13]. When activated, STAT3 is capable of mediating several critical signaling pathways, including proliferative, survival, and metastasis, and can modulate a variety of genes involved in the cell cycle (c-Myc and cyclin D1), anti-apoptosis (Bcl-2 and survivin), angiogenesis (VEGF), and metastasis (MMP-9, EMT) [35]. Here, we observed that PGG mediated the inhibition of STAT3 phosphorylation as evidenced by the suppression of the STAT3-regulated gene products, including the VEGF and Bcl-2, in PGG-treated CAL27 and FaDu cells. In addition, in the present study, we found cisplatin-induced STAT3 activation, but PGG blocked the activation dose-dependently when combined with cisplatin in both CAL27 and FaDu cells. These results agreed with the fact that DNA adduct is a molecular mechanism for cisplatin [3]. It is essential that the PGG and cisplatin action mechanisms are not overlapping, which is a desirable characteristic of synergistic combination therapy. We already studied the interactions of PGG on the SH2 domain of STAT3 receptor by computational simulation with molecular docking, molecular dynamic, and MM-PBSA techniques. The computational results strongly correlated with the Western blot experiment, showing that PGG can stably bind to the SH2 domain. The binding stability is mostly contributed by hydrogen bonds, especially Glu612, Ser636, Glu638, and Arg609 residues, and van der Waals interaction of Val637 and Pro639. In addition, energy decomposition per residue demonstrated the favored interaction of PGG with key amino acid residue Arg609, preventing the activation of STAT3 by phosphopeptide-containing pTyr705 peptide. These findings suggested that PGG inhibits the activation of STAT3 and is responsible for inhibiting cell proliferation and apoptosis induction induced by treatment with PGG and PGG plus cisplatin. The phosphatidylinositol-3 kinase (PI3K)/Akt pathway plays a crucial role in survival when cancer cells are exposed to different kinds of apoptotic stimuli and are also associated with cancer progression, migration, and involvement in drug resistance [36]. The Akt activation may lead to cell survival and inhibit apoptosis via the phosphorylation of the Bcl-2-associated agonist of cell death (Bad) and the caspase cascade pathway [37]. PGG has been reported to inhibit breast cancer cell growth by directly inhibiting Akt kinase activity [38]. In our study, PGG alone effectively suppressed Akt phosphorylation, and the combination of PGG and cisplatin potentiated the effect of cisplatin-induced Akt phosphorylation. Therefore, Akt inhibition was suspected of having a mechanism to promote cisplatin sensitivity in HNSCC. The contribution of Akt pathway inhibition to apoptosis with PGG was determined from the enhancement of cisplatin-induced apoptosis with PGG plus cisplatin. These findings suggested that PGG might inhibit the activation of the Akt pathway, leading to the acceleration of cisplatin-induced apoptosis. In addition, PGG can inhibit tumor metastasis and multiple signal pathways involved in the metastasis of breast and prostate cancers [15,18]; a future study will investigate the effects and mechanisms of PGG combined with cisplatin on tumor metastasis.

In order to approach the tumor situation in vivo more closely, a 3D tumor spheroid model and viability protocol were established. Tumor spheroids represent the architecture of solid tumors in vivo, mimicking the tumor microenvironment observed in different tumor heterogenic cells [39]. Our study agreed with other studies that 3D tumor spheroids were more resistant to treatment than 2D monolayers [40,41]. Our results showed that combination therapy of PGG and cisplatin maintained a strong cytotoxic effect in the 3D cell culture and was significantly more potent than the results of both monotherapies (Figure 8). These data demonstrated that the novel combination of PGG and cisplatin was effective against the monolayer culture of HNSCC and in 3D tumor spheres. Moreover, it is known that tumor-surrounding cells, including fibroblasts and immune cells, impact the response of tumor cells to treatment, which is a point that is usually not considered in 2D cultures [42]. However, adding different cell types and thus heterogeneity to 3D models would also add complexity, making it difficult to answer the question. Therefore, we decided here to focus on culturing conditions only (2D vs. 3D) to investigate the ability of PGG to synergize the anti-cancer effects of cisplatin in HNSCC cancer cell lines. Thus, if these novel combination treatment results are transferred to the clinical setting, it will become necessary to work with models as close to the patient’s situation as possible.

## 4. Materials and Methods

### 4.1. Chemicals and Reagents

Eagle’s Minimum Essential Medium (EMEM), Roswell Park Memorial Institute (RPMI) 1640 medium and Dulbecco’s Modified Eagle’s Medium/Nutrient Mixture F-12 (DMEM/F-12) were purchased from Caisson Lab (Smithfield, UT, USA). The cell culture products, including Trypsin-EDTA, fetal bovine serum (FBS), penicillin, and streptomycin, were purchased from Gibco Thermo Fisher Scientific (Waltham, MA, USA). 3-(4,5-dimethylthiazol-2-yl)-2,5-diphenyltetrazolium bromide (MTT), bovine serum albumin (BSA), and standard Penta-galloyl-β-D-glucose hydrate (PGG) were purchased from Sigma-Aldrich (St. Louis, MO, USA). The additional growth factors for cell culture, such as human insulin, epidermal growth factor, basic fibroblast growth factor, and hydrocortisone, were obtained from Sigma-Aldrich (St. Louis, MO, USA). The annexin V-FITC/propidium iodide (PI) apoptosis detection kit was also purchased from Sigma-Aldrich (St. Louis, MO, USA). PMSF and cocktail protease inhibitors were purchased from Hi Media Laboratories (Marg, Mumbai, India). Primary antibodies against total Akt, phosphorylated Akt (Ser473), total STAT3, phosphorylated STAT3 (Tyr705), cleaved-caspase 3, Bcl2, cleaved-PARP, and GAPDH, as well as horseradish peroxidase-labeled secondary antibodies, were purchased from Merck (Merck, Darmstadt, Germany).

### 4.2. PGG Isolation and High-Performance Liquid Chromatography (HPLC) Quantification

PGG was isolated from the seeds of *Bouea macrophylla* Griffith as described previously [13]. In brief, a crude ethanolic extract of *Bouea macrophylla* seed (MPSE) was prepared at 1 g/L in deionized water and stored at −20 °C in a freezer for 3 h, and then cooled to 4 °C by air exposure at room temperature for another 3 h. After that, the cold solution was collected and filled into a 50 mL centrifuge tube and centrifuged at 2500× *g* for 20 min. The pellets were washed with 50 mL cold water and centrifuged at 2500× *g* for 20 min for five cycle times. Next, the PGG pellets were collected and lyophilized to powder (weight = 0.114 g; yield = 11.4%) and kept in a desiccator at room temperature for further study. PGG purity was 97% quantified using a Shimadzu LC-20AD Prominence Liquid Chromatograph system equipped with an SPD-M20A Prominence Diode Array Detector (Shimadzu, Nakagyo-Ku, Kyoto, Japan) according to a previously well-established and validated method [43]. For the experiment, PGG was dissolved in 1 mL of deionized water and sonicated for 30 min to make a stock solution of 1 mg/mL (1.06 mM), while cisplatin was prepared as a 1 mg/mL (3.32 mM) stock in 0.9% sodium chloride (NaCl). Both PGG and cisplatin were filtered through a 0.22-µm membrane, aliquoted, and stored at −20 °C until further use. The drugs were then diluted in a complete medium to provide a final working concentration.

### 4.3. Cell Lines and Cell Culture Conditions

Human head and neck squamous cell carcinoma CAL27 (CRL-2095™), FaDu (HTB-43™), and normal mouse fibroblast NCTC clone 929 (L929) cell lines were purchased from American Type Culture Collection (Manassas, VA, USA). The cancer cell lines CAL27 and FaDu were cultured in Eagle’s Minimum Essential Medium (EMEM) containing 10% FBS, 2 mM l-glutamine, 100 U/mL penicillin, and streptomycin. L929 were maintained in Roswell Park Memorial Institute (RPMI) 1640 medium supplemented with 10% FBS, 2 mM l-glutamine, and 100 U/mL penicillin and streptomycin. All cell lines were cultivated at 37 °C in a humidified incubator with 5% CO_2_.

### 4.4. Cell Viability Assay

Cells were seeded onto 96-well plates at 5 × 10^3^ cells/well density and allowed to grow overnight in an incubator at 37 °C. Cells were treated with PGG (0–100 µg/mL or 0–106.3 µM), cisplatin (0–15 µg/mL or 0–49.8 µM), or with a combination of the two compounds with a fixed concentration of PGG (10, 20, 30 µg/mL or 10.6, 21.3, 31.9 µM) and various concentrations of cisplatin (0–15 µg/mL or 0–49.8 µM). After treatment, cells were cultured for 48 h, and cell viability was assessed by a 3-(4,5-dimethylthiazol-2-yl)-2,5-diphenyltetrazolium bromide (MTT) assay. In brief, After the treatment period, 20 µL of the MTT solution (final concentration of 0.1 mg/mL) were added to the cells and then incubated at 37 °C for 4 h. The Formazan crystal product was dissolved in 100 µL of dimethyl sulfoxide (DMSO). The absorbance of formazan solution was measured at 560 nm using a microplate reader (BioTek^TM^ Eon^TM^ microplate reader, Winooski, VT, USA). The absorbance of the treated cells was divided by that of the control cells to determine relative cell viability. Three independent experiments were used to determine the half-maximal inhibition concentration (IC_50_) using OriginPro version 2018 software (Origin Lab, Northampton, MA, USA). The combination index (CI) was determined using the Chou–Talalay method and the software package CompuSyn (Biosoft, Ferguson, MO, USA). Then, we employed a Fa-CI plot (Fa–fraction affected) analysis to determine whether the interactions between two compounds were additive, synergistic, or antagonistic. A CI value of less than one was defined as synergism [44].

### 4.5. Colony-Formation Assay

Human head and neck squamous cell carcinoma CAL27 and FaDu cell lines were seeded in 6-well plates and allowed to grow overnight, then treated with PGG, cisplatin, or a combination of the two drugs for 48 h. Cells were maintained for 14 days to allow colonies to form. After that, colonies were fixed in fixation solution (3:1 of methanol/acetic acid) for 30 min at room temperature and stained with 1% crystal violet. Cell colonies with more than 50 cells were imaged and counted using an inverted microscope (ECLIPSE Ts2, Nikon, Tokyo, Japan).

### 4.6. Apoptosis Assay in Monolayer Tumor Cells by Annexin V-FITC/PI Double Staining

Cells were seeded into a 6-well plate for 24 h to allow attachment before treatment with varying concentrations of either PGG, cisplatin, or a combination of the two drugs.

After treatment for 48 h, cells were detached with a trypsin-EDTA solution and then washed with 1× binding buffer. The collected cells were dual stained with the annexin V-FITC and PI for 20 min at room temperature in complete darkness, following the manufacturer’s instructions. The stained cells were immediately analyzed by flow cytometry (CytoFLEX, Beckman Coulter, Brea, CA, USA). The flow cytometric data was then analyzed using the FlowJo^TM^ software version 10 (Becton, Dickinson & Company, Buena, NJ, USA).

### 4.7. Scratch Wound Healing Assay

CAL27 and FaDu cells (5 × 10^5^ cells/well) were grown in a 6-well plate to form a confluent monolayer. A wound was then created using a sterile 200 µL micropipette tip, and the detached cells were removed by washing with phosphate buffer saline. The cells were then treated with PGG, cisplatin, or a combination of the two drugs and further incubated in EMEM medium with 0.5% FBS for 48 h. The wound gap was observed and photographed using phase-contrast microscopy (Nikon, ECLIPSE Ts2, Tokyo, Japan). The images were analyzed using ImageJ software 1.52v version (National Institutes of Health, Bethesda, MD, USA) to measure the width of the scratch.

### 4.8. Transwell Migration Assay

Cell migration assays were performed using Transwell chambers (8 µm pore size, Corning Co., Corning, NY, USA). CAL27 and FaDu cells were suspended in a serum-free medium at a density of 1 × 10^5^ cells/mL. Then, 200 µL of cell suspension containing PGG, cisplatin, or a combination of the two drugs was added into the upper Transwell chamber. The lower compartments were filled with 600 µL of medium with 20% FBS. After incubation for 24 h, cells on the upper layer of the chamber were excluded with a cotton swab, and the cells that had migrated through to the underside of the insert membranes were fixed with 4% paraformaldehyde for 10 min and stained with 1% crystal violet for 20 min. Cells in five separate microscope fields were counted and captured under an inverted microscope (ECLIPSE Ts2, Nikon, Tokyo, Japan).

### 4.9. Western Blot Analysis

After treatment, cells were lysed for 30 min on ice with CelLytic^M^ lysis solution (Sigma-Aldrich, St. Louis, MO, USA) and a 1% protease inhibitor cocktail to extract the proteins from the cells. The total protein concentrations were determined by the Bradford assay (Sigma-Aldrich). An equal amount of protein samples (15 µg) was separated on NUPAGE^TM^ 4–12% Bis-Tris Gels (Thermo Fisher Scientific, Waltham, MA, USA). The separated proteins from the gel were transferred and deposited into a PVDF membrane (Millipore, St. Louis, MO, USA). The whole transferred membranes were cut at the appropriate molecular weight range of specific protein and blocked for 1 h in 5% nonfat dry milk in Tris-buffered saline with Tween and incubated overnight with specific primary antibodies against total Akt, phosphorylated Akt, total STAT3, phosphorylated STAT3, cleaved-caspase 3, cleaved-PARP, Bcl-2, VEGF, as well as GAPDH. Then, membranes were washed, followed by incubation with appropriate horseradish-peroxidase-labeled secondary antibodies (at a dilution of 1:10,000) for 1 h at room temperature. Signals were developed using the enhanced chemiluminescence assay (Millipore Corporation) and exposed to film. The images were scanned, and the intensity of each band was captured using ImageMaster 2D platinum version 5.0 (GE Healthcare Amersham Bioscience, Chicago, IL, USA). The protein content was normalized to GAPDH.

### 4.10. Three-Dimensional (3D) Tumor Model and Drug Response Assays

Human head and neck squamous cell carcinoma CAL27 and FaDu cell lines were grown in 3D cultures to form multicellular tumor spheroids. First, single cells at 1 × 10^3^ cells/well were suspended in spheroid media (serum-free DMEM/F-12 media supplemented with 1% BSA, 5 µg/mL insulin, 25 ng/mL basic fibroblast growth factor, 25 ng/mL epidermal growth factor, and 0.5 µg/mL hydrocortisone) and plated in a Matrigel Matrix Growth Factor Reduced (BD Biosciences) precoated 96-well ultra-low attachment culture plate for 48 h at 37 °C in 5% CO_2_ to form spheroid tumors. Next, spheroids were treated by applying monotherapy and combination therapy. Pictures of spheroids were taken with an inverted phase-contrast microscope (ECLIPSE Ts2, Nikon, Tokyo, Japan) every other day for 9 days. Spheroid dimensions were quantified using ImageJ 1.52v software (National Institutes of Health, USA). After 9 days of growth, spheroids were moved separately to a single well of a 96-well plate and cell viability was determined using an ATPlite^TM^ luminescence assay (PerkinElmer, Waltham, MA). The luminescence signal was measured using a luminescent microplate reader (SpectraMax^®^ i3x multi-mode microplate reader, Molecular Devices, LLC, San Jose, CA, USA).

### 4.11. Cell Viability (Live/Dead) in 3-Dimensional Tumor Model by Propidium Iodide (PI)/Acridine Orange (AO) Staining

Multicellular tumor spheroids were treated with PGG, cisplatin, or a combination of the two drugs for 96 h. Then, spheroids were washed with PBS, after which a solution containing PI/AO was added and incubated for 30 min. The stained cells were immediately visualized and imaged under an inverted fluorescence microscope (ECLIPSE Ts2, Nikon, Tokyo, Japan). The differentiation between viable and dead cells was based on the difference in dye permeability into the intact cell membrane. Green cells represented viable cells and were stained only with AO; green and orange cells with condensed chromatin represented an apoptotic cell and were stained with AO and PI; finally, necrotic cells were red and stained with PI. Six spheroids per treatment were imaged to ensure that the data obtained were representative. All images were equally processed, adjusting the contrast and saturation associated with each fluorescence channel.

### 4.12. Molecular Docking

The signal transducer and activator of transcription 3 (STAT3) play important roles in the multidrug resistance of cells. This protein was utilized to clarify the mechanism of action of pentagalloyl glucose in the drug combination study. The protein structures of STAT3 in complex with inhibitor SI109 (PDB ID: 6NUQ) were retrieved from a protein databank (https://www.rcsb.org/, accessed on 30 November 2021). Protein structures were prepared with BIOVIA Discovery Studio 2020 software (BIOVIA Discovery Studio Visualizer, version 21.1.0.20298, San Diego, CA, USA). The water molecules, ions, and ligands were removed. Missing residues were added using the Modeller 10 package [45], and the standard protonation state was calculated at pH 7.4 with the ProPka server [46]. 3D structures of ligands were generated by GaussView 6 software [47]. The structures were optimized via DFT calculation using B3LYP/6-311G++(d, p) as the basis set for calculation with the Gaussian 16 package [48]. Ligands were further prepared using the AutoDock Tool 1.5.6 package by merging non-polar hydrogens, assigning Gasteiger charges, and converting to pdbqt format. All molecular docking experiments were performed on the HP Z8 G4 workstation with an Intel^®^ Xeon^®^ Scalable Processor comprising 20 physical processors running on the Ubuntu 20.04 operating system. AutoDock Vina software was used for docking simulation [49]. The docking methods were validated by redocking of the co-crystalized ligand into the binding pocket. The grid parameters were generated using the AutoDock Tool 1.5.6 package. The grid size was defined as 40 × 58 × 40 (x, y, z) with a grid spacing of 0.375 Å. The grid box was generated by covering the active site of the SH2 domain on chain A at 13.619, 54.024, −0.083 along x, y, and z dimensions. The best binding pose was visualized, and protein-ligand interactions were analyzed with BIOVIA Discovery Studio 2020.

### 4.13. Molecular Dynamic Simulations

All molecular dynamic simulations were conducted on an HP Z8 G4 workstation and were accelerated by NVIDIA^®^ Quadro^®^ P4000 GPU. The simulations of protein-ligand complexes were achieved via the GROMACS 5.1.4 package [50] using GROMOS 54A7 force field [51] for the generation of topology and coordinate files. The best binding poses of ligands on receptors were selected to further investigate their dynamic. Topologies of ligands were generated by Automated Topology Builder (ATB) and the Repository Version 3.0 server, available at https://atb.uq.edu.au/, accessed on 12 December 2021 [52]. The protein-ligand complex was placed in the dodecahedral box, maintaining a distance of 1.0 nm between the solutes and the side of the box, followed by the solvation of water molecules using the SPC model. Sodium (Na^+^) and chloride (Cl^−^) ions were added for neutralization. The system minimized the energy using the steepest descent algorithm with a tolerance value of 10 kJ/mol/nm. Equilibration of the system was restrained by a constant number of particles, volume, and temperature (NVT) and a constant number of particles, pressure, and temperature (NPT) ensemble for 1000 ps. In the equilibration steps, only the solvent molecules were allowed free movement while other atoms were restrained. Long-range electrostatic interactions were calculated with the particle mesh Ewald method with a 12 Å cut-off. Temperature and pressure couplings were calculated with the Modified Berendsen thermostat and Parrinell–Rahman barostat, respectively. Finally, the equilibrated systems were entered into a production state without restraints for 50 ns at 310 K and 1 bar atmospheric pressure. Root mean square deviation (RMSD) was calculated using a generated trajectory of the simulation. Hydrogen bonds were analyzed using a built-in script on GROMACS software.

### 4.14. Free Binding Energy Calculations

Dynamic profiles of protein-ligand complexes were further investigated through the binding energies between protein and ligand. The Molecular Mechanic Poisson–Boltzmann Surface Area (MM-PBSA) method of the g_mmpbsa package as developed by Kumari et. al. was used for calculation [53]. Then, 1000 snapshots from the equilibrated region of trajectory were extracted every 10 ps. The total free binding energy (ΔG_bind_) was calculated according to the following equations:

ΔG_bind_ = ΔG_complex_ − (ΔG_protein_ − ΔG_ligand_), (1)

ΔG_bind_ = ΔE_MM_ + ΔE_polar_ + ΔE_SASA_ – TΔS, (2)

ΔE_MM_ = ΔE_elect_ + ΔE_vdw._ (3)

The vacuum potential energy (ΔE_MM_) was calculated by the molecular mechanic (MM) force field to provide electrostatic (ΔE_elect_) and van der Waals (ΔE_vdw_) energies based on Coulomb potential and Lennard–Jones potential functions, respectively. Polar solvation energy (ΔE_polar_) was calculated based on the Poisson–Boltzmann equation. Non-polar solvation energy (ΔE_SASA_) was calculated based on the solvent-accessible surface area (SASA) function. Decomposition energy terms were computed by python scripts MmPbSaDecomp.py and MmPbSaStat.py as available in the g_mmpbsa package.

### 4.15. Statistical Analysis

Data obtained from at least three independent experiments are expressed as the mean ± standard deviations (SDs). The data were analyzed for differences in the mean values by one-way ANOVA followed by post-hoc analysis using IBM^®^ SPSS^®^ Statistics Subscription software (IBM Corp. in Armonk, NY, USA). A value of *p* < 0.05 was considered statistically significant.

## 5. Conclusions

In summary, our findings indicated that the combination therapy of PGG and cisplatin produced a synergistic effect on the inhibition of HNSCC cancer cell viability and induced apoptosis in 2D and 3D models. Moreover, this combination also enhanced the inhibition of HNSCC cancer cell migration and invasion. The mechanism of the anti-cancer effects of combined PGG and cisplatin was demonstrated to occur, at least partially, via inhibiting the STAT3/Akt signaling pathway. The computational approaches strongly supported the inhibition of STAT3 by PGG. The present results suggested that PGG may be a promising adjunct drug when used with cisplatin for a practical therapeutic approach to head and neck cancer. Although the combined therapy of PGG and cisplatin had an apparent inhibitory effect on HNSCC cancer cell proliferation and growth in vitro and ex vivo, these effects have not yet been confirmed in animal models. Further research and clinical trials are warranted to fully elucidate the effects of this combined therapy on the HNSCC cancer model.

## Figures and Tables

**Table 1:** Summary of cisplatin IC_50_ (µg/mL), combination index (CI) values, and dose reduction index (DRI) of PGG and cisplatin combination treatment in HNSCC cells.

Treatment	CAL27			FaDU		
	IC_50_ (µg/mL)	CI at IC_50_	DRI	IC_50_ (µg/mL)	CI at IC_50_	DRI
Cisplatin alone	5.4 ± 0.4			7.6 ± 1.4		
Cisplatin with 10 µg/mL PGG	4.0 ± 0.1	1.2	1.35	5.4 ± 0.9	1.13	1.41
Cisplatin with 20 µg/mL PGG	1.4 ± 0.2	0.64	3.86	3.6 ± 1.7	0.81	2.11
Cisplatin with 30 µg/mL PGG	0.8 ± 0.6	0.51	6.75	1.2 ± 0.6	0.62	6.33

Different concentrations of PGG were employed to study the effect on IC_50_ of cisplatin. Drug interactions were calculated by variable ratios of drug concentrations and mutually non-exclusive equations using Calcusyn. The CI value >1 indicates antagonism, CI value = 1 indicates additive, and CI value < 1 indicates synergism. DRI was measured by comparing the doses when used as a single treatment or combination. PGG, pentagalloyl glucose; CI, combination index; DRI, dose reduction index; IC_50_, half-maximal inhibitory concentration.

**Table 2:** Most contributed hydrogen bonding interactions between PGG and STAT3.

No.	Hydrogen Bonding *	Distance (Å)	Occupancy (%)
1	Glu612-C=O····HO-PGG	1.758 ± 0.181	92.91
2	Glu612-C=O····HO-PGG	1.669 ± 0.152	88.62
3	Glu612-C=O····HO-PGG	1.674 ± 0.156	85.43
4	Glu612-C=O····HO-PGG	1.665 ± 0.156	80.14
5	Ser636-NH····O=C-PGG	2.416 ± 0.404	60.08
6	Ser636-C=O····HO-PGG	2.463 ± 0.870	40.92
7	Glu638-C=O····HO-PGG	2.785 ± 0.538	23.15
8	Arg609-NH·····O-PGG	2.450 ± 0.382	19.66
9	Arg609-NH····O-PGG	2.653 ± 0.597	18.56
10	Arg609-NH····O-PGG	2.486 ± 0.372	10.58
11	Ile634-C=O····HO-PGG	2.706 ± 0.549	3.49

* Hydrogen bonds are determined by the donor-acceptor angle > 120° and distance of < 3.5 Å.

**Table 3:** Molecular energetic terms of interactions between PGG and STAT3 receptor.

Terms	Energy (kcal/mol)
van der Waals (ΔE_vdw_)	−46.858 ± 4.360
electrostatic (ΔE_elect_)	−47.356 ± 4.681
polar solvation (ΔE_polar_)	72.616 ± 7.008
SASA (ΔE_SASA_)	−5.861 ± 0.3566
binding energy (ΔG_bind_)	−27.938 ± 5.224

**Table 4:** Per residue energy contribution of PGG interacting with the binding site of STAT3.

Residue	Energy (kcal/mol)			
	Potential Energy (ΔE_MM_)	Polar Solvation (ΔE_polar_)	SASA (ΔE_SASA_)	Binding Energy (ΔG_bind_)
Lys531	−0.916 ± 0.017	−1.721 ± 0.024	−0.001 ± 0.000	−0.806 ± 0.015
Lys557	−1.522 ± 0.036	1.023 ± 0.075	−0.075 ± 0.002	−0.574 ± 0.057
Arg609	0.515 ± 0.025	−0.890 ± 0.052	−0.142 ± 0.001	−0.518 ± 0.043
Ser613	−0.091 ± 0.014	−0.258 ± 0.017	−0.146 ± 0.002	−0.495 ± 0.016
Lys615	−0.882 ± 0.014	0.034 ± 0.011	−0.001 ± 0.000	−0.848 ± 0.010
Thr620	−0.928 ± 0.008	0.457 ± 0.009	−0.054 ± 0.001	−0.525 ± 0.010
Thr622	−0.327 ± 0.005	−0.588 ± 0.006	−0.009 ± 0.000	−0.923 ± 0.006
Trp623	−0.735 ± 0.007	0.288 ± 0.004	−0.068 ± 0.001	−0.514 ± 0.007
Ile634	−0.793 ± 0.009	0.435 ± 0.010	−0.075 ± 0.001	−0.433 ± 0.009
Ser636	−4.245 ± 0.021	4.300 ± 0.019	−0.349 ± 0.001	−0.296 ± 0.023
Val637	−2.886 ± 0.012	1.632 ± 0.013	−0.147 ± 0.001	−1.402 ± 0.014
Pro639	−1.269 ± 0.007	0.015 ± 0.002	−0.118 ± 0.001	−1.372 ± 0.007

## Data Availability

Data are contained within the Article and Supplementary Materials.

## Supplementary Materials

The following supporting information can be downloaded at: https://www.mdpi.com/article/10.3390/ph15070830/s1, Figure S1: The cell proliferation inhibitory effect of PGG on normal mouse fibroblasts L929 cells in the 2D culture determined by the MTT assay; Figure S2: Superimposition of redocked (blue) and co-crystalized (red) ligands of SI109 inhibitor for validation of docking protocol of STAT3 receptor; Figure S3: Two-dimensional protein-ligand interactions of STAT3-PGG complex from molecular docking simulation; Figure S4: The uncropped western blot images corresponding to Figure 3d; Figure S5: The uncropped western blot images corresponding to Figure 3e; Figure S6: The uncropped western blot images corresponding to Figure 5a; Figure S7: The uncropped western blot images corresponding to Figure 5c.
