# Supplementary material for: Pentagalloyl Glucose and Cisplatin Combination Treatment Exhibits a Synergistic Anticancer Effect in 2D and 3D Models of Head and Neck Carcinoma
Source: Pharmaceuticals (Basel). 2022 Jul 4;15(7):830. doi: 10.3390/ph15070830 (PMC9317773; doi:10.3390/ph15070830)
Supplement: Supplementary file 1 [file pharmaceuticals-15-00830-s001.zip › pharmaceuticals-1787984-supplementary.pdf]

pharmaceuticals

Supplementary Materials

Pentagalloyl Glucose and Cisplatin Combination Treatment Exhibits a Synergistic Anticancer Effect in 2D and 3D Models of Head and Neck Carcinoma

Jiraporn Kantapan[1,2], Nuttawadee Intachai[2], Nopawit Khamto[3,4], Puttinan Meepowpan[3,5], Padchanee Sangthong[3], Kittichai Wantanajittikul[2], Nathupakorn Dechsupa[1,2,*] and Imjai Chitapanarux[6*]

[1]Molecular Imaging and Therapy Research Unit, Faculty of Associated Medical Sciences, Department Radiologic Technology, Chiang Mai University, Chiang Mai 50200, Thailand
[2]Faculty of Associated Medical Sciences, Department Radiologic Technology, Chiang Mai University, Chiang Mai 50200, Thailand
[3]Faculty of Science, Department of Chemistry, Chiang Mai University, Chiang Mai 50200, Thailand
[4]Graduate School, Chiang Mai University, Chiang Mai 50200, Thailand
[5]Center of Excellence in Material Science and Technology, Chiang Mai University, Chiang Mai 50200, Thailand
[6]Faculty of Medicine, Department of Radiology, Division of Radiation Oncology, Chiang Mai University, Chiang Mai 50200, Thailand
*** Correspondence:**
Nathupakorn Dechsupa
Nathupakorn.d@cmu.ac.th
Imjai Chitapanarux
imjai.chitapanarux@cmu.ac.th

Supplementary Materials: Figure S1: The cell proliferation inhibitory effect of PGG on normal mouse fibroblasts L929 cells in the 2D culture determined by the MTT assay; Figure S2: Superimposition of redocked (blue) and co-crystalized (red) ligands of SI109 inhibitor for validation of docking protocol of STAT3 receptor; Figure S3: Two-dimensional protein-ligand interactions of STAT3-PGG complex from molecular docking simulation; Figure S4: The uncropped western blot images corresponding to Figure 3d; Figure S5: The uncropped western blot images corresponding to Figure 3e; Figure S6: The uncropped western blot images corresponding to Figure 5a; Figure S7: The uncropped western blot images corresponding to Figure 5c.

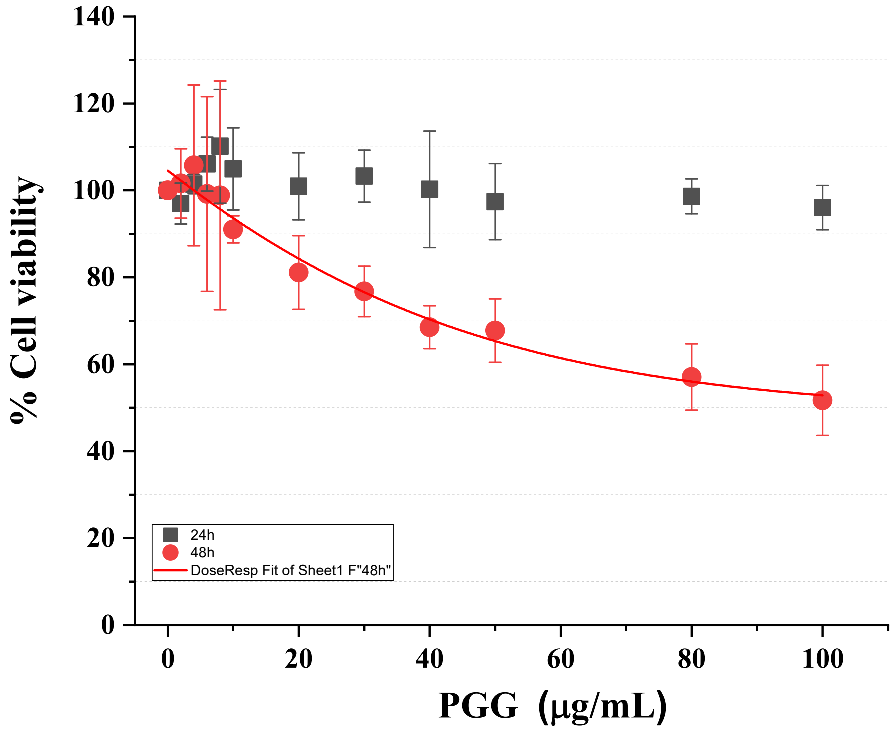

Supplementary Figure S1. The cell proliferation inhibitory effect of PGG on normal mouse fibroblasts L929 cells in the 2D culture determined by the MTT assay.

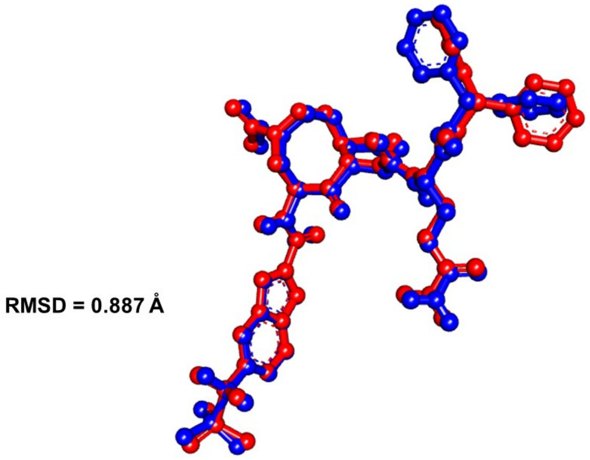

Supplementary Figure S2. Superimposition of redocked (blue) and co-crystalized (red) ligands of SI109 inhibitor for validation of docking protocol of STAT3 receptor.

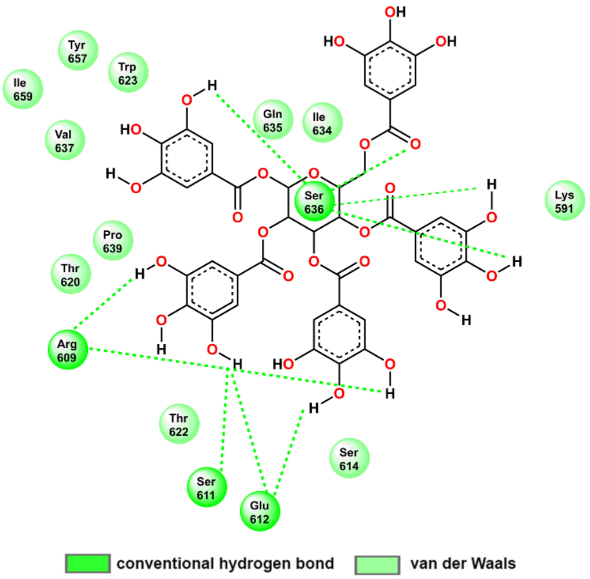

Supplementary Figure S3. Two-dimensional protein-ligand interactions of STAT3-PGG complex from molecular docking simulation.

pharmaceuticals

MDPI

Figure 3d

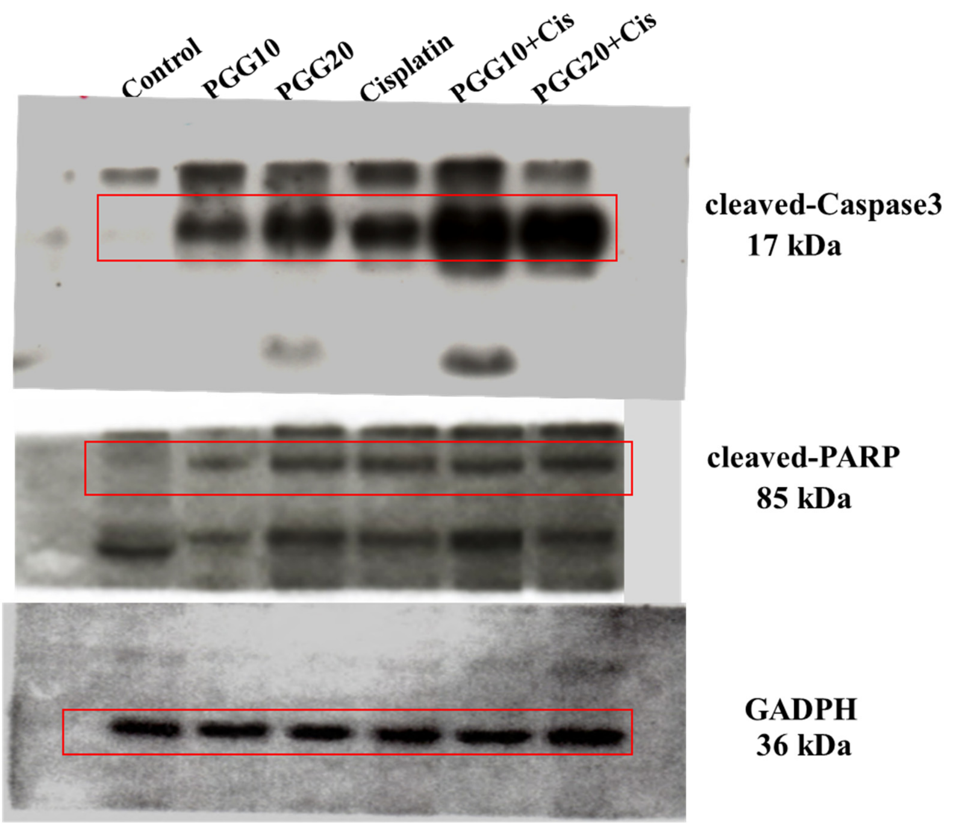

Supplementary Figure S4. The uncropped Western blot images corresponding to Figure 3d showing all the bands. Red boxes indicate the samples of interest.

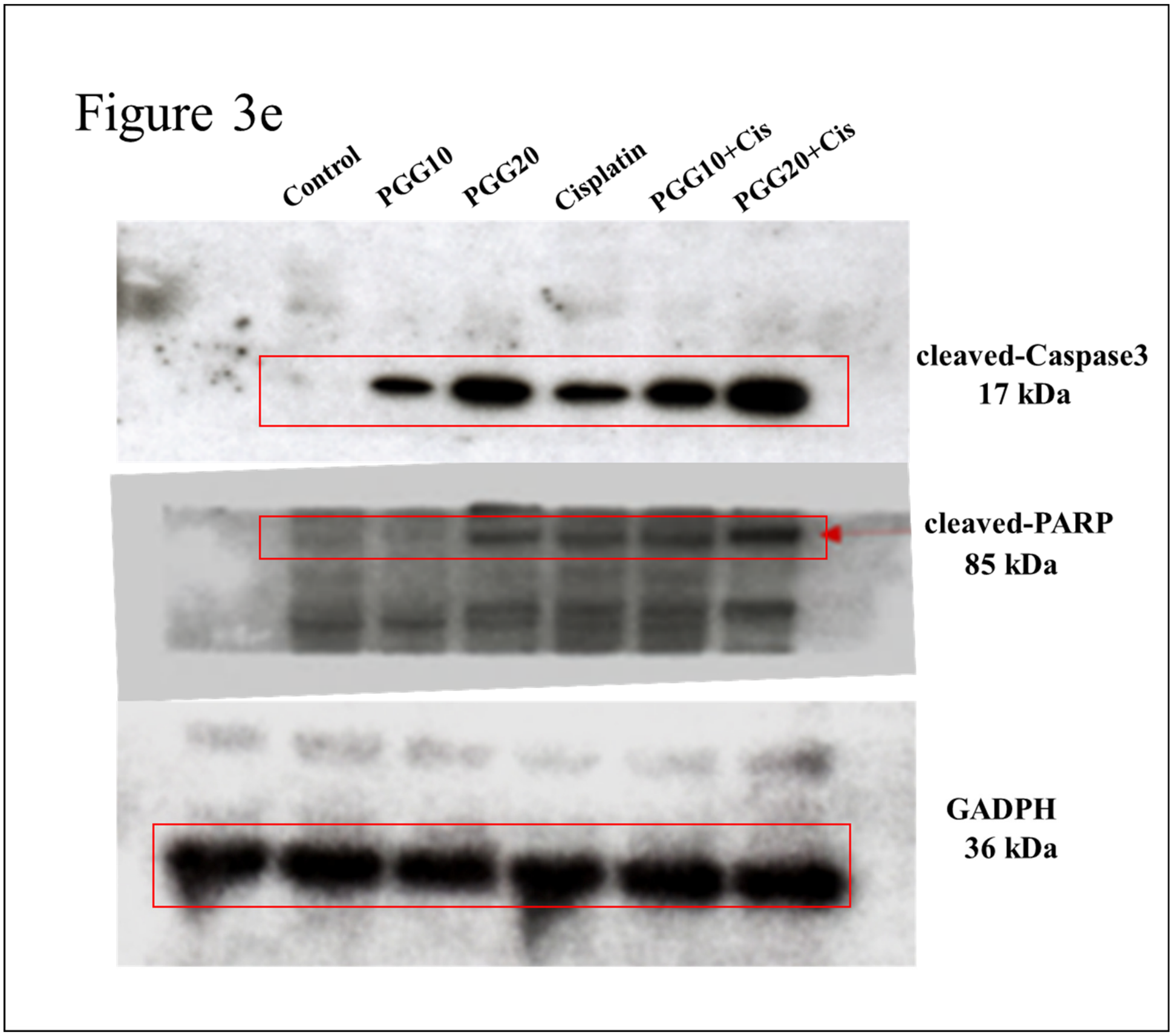

Supplementary Figure S5. The uncropped Western blot images corresponding to Figure 3e showing all the bands. Red boxes indicate the samples of interest.

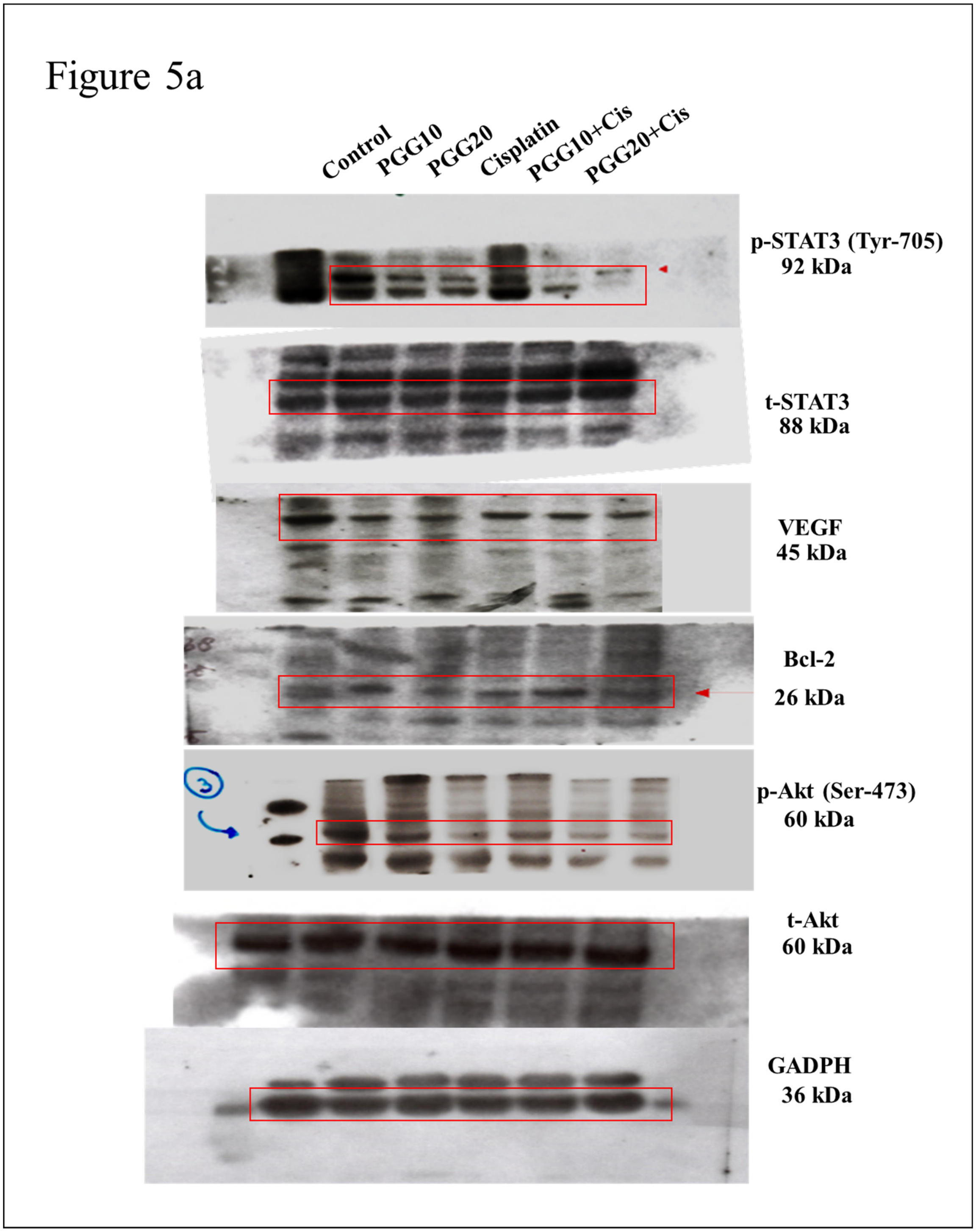

Supplementary Figure S6. The uncropped Western blot images corresponding to Figure 5a showing all the bands. Red boxes indicate the samples of interest.

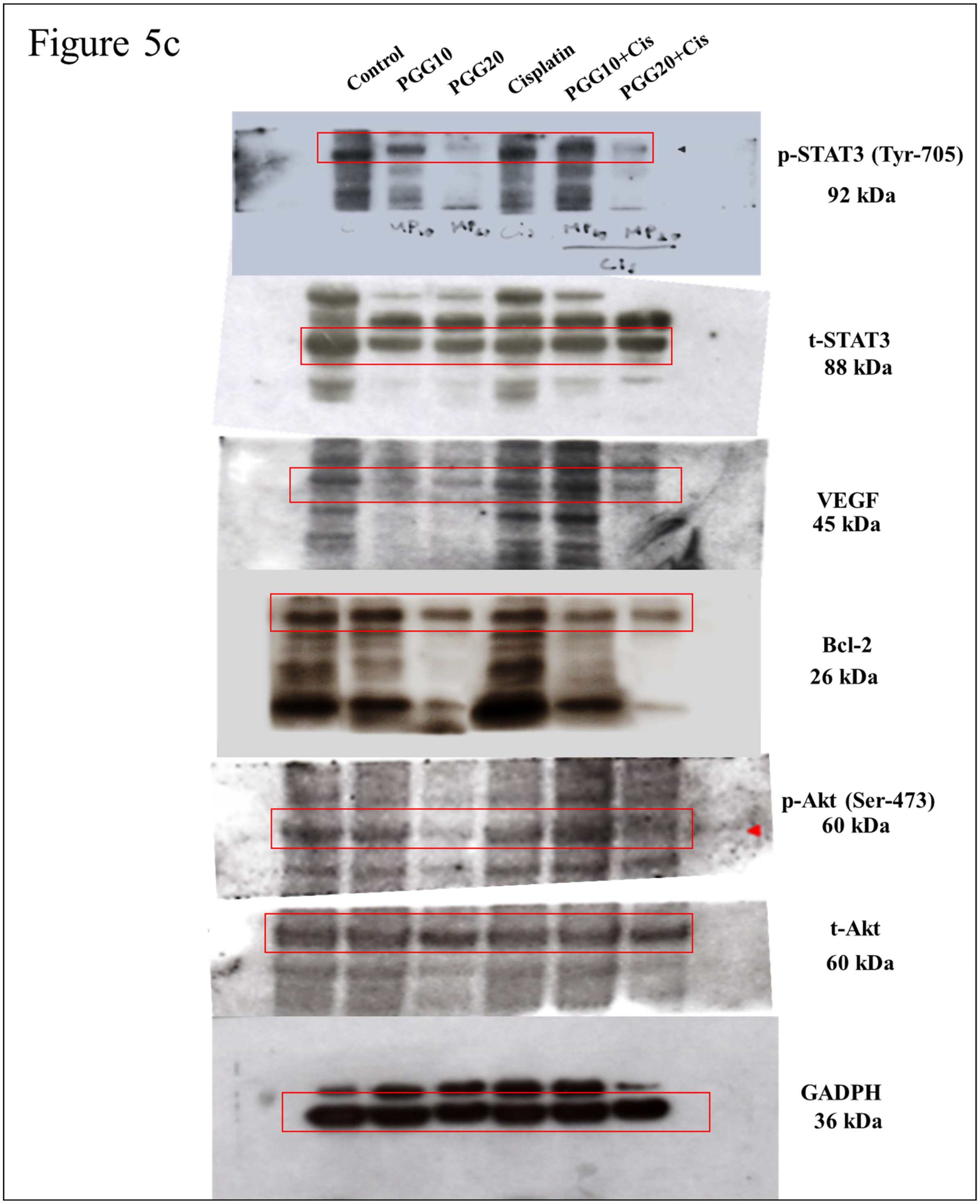

Supplementary Figure S7. The uncropped Western blot images corresponding to Figure 5c showing all the bands. Red boxes indicate the samples of interest.
